# 

**Published:** 1909-12-15

**Authors:** 


### ANNOUNCEMENTS.

Institute of Dental Pedagogics. The seventeenth annual meeting of the Institute of Dental Pedagogics will be held at the King Edward Hotel, Toronto, Canada, December 28th, 29th and 30th, 1909.

The Institute is composed of dental teachers of the United States and Canada. An excellent program has been prepared, and matters of vital interest in the advancement of dental education are under discussion. Interesting and valuable teaching methods and appliances will be exhibited.

Dental teachers, examiners and ethical practitioners, who are interested in the advancement of dental education are cordially invited.

Further particulars can be had from the Chairman of the Executive Board, Dr. H. E. Friesell, Dental Department, University of Pittsburgh, Pittsburgh, Pa.

IOWA STATE BOARD OF EXAMINERS. Examination of candidates for license to practice dentistry in Iowa will be held at Des Moines, Iowa, January 10th, 1910, at 9 A. M. For information write to Dr. E. D. Brower, Secy., Le Mars, Iowa.

PATENT DECISION. By a decision in the United States Circuit Court in New York, Dr. Angle has sustained his right to the patented friction nut on the regulating arch. The suit was brought by Dr. Angle to prevent the manufacture of a similar device by Julius Aderer of New York City. There can be no question that Aderer's device was a slight modification only of Angle's, and Dr. Angle is to be congratulated on the establishment of his rights.

G. V. BLACK BANQUET. The Chicago Odontographic Society will give a testimonial banquet to Dr. G. V. Black at Chicago, January 29th in the Gold Room of Congress Hotel. There is no man whom the dentists of the West more unaminously like to honor, and we predict this will be a rare occasion and a feast of reason and flow of soul will certainly characterize this occasion.

INDIANA STATE BOARD OF EXAMINERS. Candidates for to practice in Indiana will be examined at a meeting held at Indianapolis, in the State House, January 10th to 14th, 1910. For information address Dr. F. R. Henshaw, Secy., 507 Pythian Building, Indianapolis, Ind.

HARVARD DENTAL SCHOOL'S NEW BUILDING. On December seventh the new building of this school was formally dedicated by appropriate exercises. The new building is located adjoining the new medical building and is fully equipped with modern facilities. The profession will rejoice in this, another added help to better educational facilities.

